# Media portrayal of vaccine: a content analysis of Vietnam online news about a pentavalent vaccine in the Expanded Program of Immunization.

**DOI:** 10.12688/wellcomeopenres.18457.1

**Published:** 2022-11-02

**Authors:** Nguyen Thanh Ha, Nguyen Thi Yen Chi, Jennifer Van Nuil, Louise Thwaites, Mary Chambers

**Affiliations:** 1 Oxford University Clinical Research Unit, Hochiminh, Vietnam; 2 Global Health Bioethics Network, Oxford, UK; 3 Department of International Health, Maastricht University, Maastricht, The Netherlands; 4 Centre for Tropical Medicine and Global Health, Oxford, UK

**Keywords:** Vaccine media, adverse events following immunization, vaccine hesitancy, childhood vaccines, Expanded Program on Immunization

## Abstract

**Background:** Vaccine hesitancy has become a prominent public health concern, particularly within the coronavirus disease 2019 (COVID-19) pandemic context. Worries about vaccine side effects are often cited as a reason for hesitancy, while media reporting about this topic plays an important role in influencing the public’s perspectives about vaccines and vaccination. In Vietnam, during 2012-2013, there were several adverse events following immunization (AEFIs) of Quinvaxem– a pentavalent vaccine in the Expanded Immunization Program, which made big headlines in the media. Such incidences have contributed to changes in vaccination policies and influenced parents’ concerns to date. This study explores the portrayal of Quinvaxem in Vietnam digital news during four periods marked by important events.

**Methods:** We performed quantitative and qualitative analysis with a coding framework to identify main content focus, sentiments towards Quinvaxem, and emotional tones in these articles.

**Results:** In total, we included 360 articles into analysis. The amount of news coverage about Quinvaxem increased after AEFIs happened, from 7 articles before AEFIs to 98 and 159 articles in the following periods when AEFIs and investigation into vaccine safety occurred. Most articles are neutral in titles (n=255/360) and content (n=215/360) towards Quinvaxem and do not convey emotional expressions (n=271/360). However, articles focusing on side effects contain negative sentiments and emotional expressions more frequently than articles of other contents while AEFIs details were conflicting across articles. Vaccine sentiments are provoked in the information about vaccine quality and safety, health authority, local delivery, and quoted vaccine opinions. Emotion-conveying elements in 89/360 articles included emotional wording and imagery and expressive punctuation.

**Conclusions**: The heterogeneity of information in online news may reinforce uncertainty about vaccine safety and decrease vaccine intention. Our results have important implications for vaccine communication, given the current plan of the Vietnamese government to roll out COVID-19 vaccination to younger children.

## Introduction

Vaccine hesitancy, defined as reluctance or refusal of one or more vaccines, has emerged as a prominent concern in public health ^1^. The World Health Organization declared vaccine hesitancy to be one of its ten threats to global health, demanding attention in its 2019 five-year strategic plan aiming to save 29 million lives ^2^. With extensive vaccination recognized by the World Health Assembly ^3^ as an important public health goal in the coronavirus disease 2019 (COVID-19) pandemic and clear evidence of the role of unvaccinated individuals in severe acute respiratory syndrome coronavirus 2 (SARS-CoV-2) transmission and health-resource utilization, understanding drivers of vaccine hesitancy is vital. Most data related to vaccine hesitancy come from high-income settings but understanding the extent and drivers behind vaccine hesitancy in low- and middle-income countries (LMICs) is essential if effective global immunization programs are to be achieved. Current data suggest significant differences in vaccine acceptance between countries, and between different vaccines offered. A recent review of global vaccine acceptance has shown that acceptance rates vary from less than 30% in some Middle Eastern countries to over 90% in Southeast Asia ^4^. Arce and colleagues ^5^ also revealed that low and middle-income countries (LMICs) in general report higher acceptance rates for COVID-19 vaccines compared to the USA and Russia, motivated mainly by interest in personal protection. The most common reason for hesitancy in LMICs relates to vaccine side effects, which may be aggravated by reporting of rare adverse events.

Information about Adverse Events Following Immunization (AEFIs) from media has particularly been documented as an important factor in influencing people’s vaccine confidence and intention ^6– 10^. When incidences of AEFIs are reported, there is often a surge in media coverage about these incidents, the vaccine, and related government policies, accompanied by a pattern of increased negative sentiments in reported information ^7, 11– 14^. Negativity about vaccines and post-vaccination reactions in media often includes misleading or biased expressions such as vaccines being ineffective, vaccines causing harm, scientific uncertainty, and inaccurate reporting of AEFIs incidences ^15– 18^. In vaccine-critical media content, case stories of AEFIs are often narrated to appeal to readers’ emotions, for example through parents’ testimony about their children being harmed by vaccines ^19^ or pictures of needles or of children’s responses to injections ^12, 20^.

Without accurate and neutral portrayal, the negative picture of vaccination painted by media reports can increase vaccine hesitancy and reduce uptake. For example, Hansen and Schmidtblaicher ^6^ found that mainstream media coverage of AEFIs of human papilloma virus (HPV) vaccine was a significant predictor of the sharp decline in HPV vaccination rates over subsequent weeks in Denmark. In Japan, newspapers’ critical coverage of HPV vaccines following some adverse reactions, in conjunction with the government’s suspension of the vaccine, also fueled public concerns and decreased uptake ^21^. Such incidents also spread to other countries through online networks and were then propagated by the global antivaccine community ^22^.

In Vietnam, extensive media attention was given to the Quinvaxem vaccine, a pentavalent vaccine, including vaccines for diphtheria, tetanus and pertussis (DTP). The vaccine has been part of the country’s national Expanded Program on Immunization (EPI) since June 2010, and its coverage is measured through the Program’s ‘DTP’ metrics. Extensive media coverage followed nine deaths occurring shortly after vaccination with Quinvaxem in 2012 and 2013, igniting widespread concerns about the vaccine ^23, 24^. The Ministry of Health (MoH) decided to suspend the use of Quinvaxem in May 2013 and requested independent investigations from different institutions. When no causal links were identified, the MoH resumed delivering Quinvaxem in the routine program in October 2013 but only allowed a maximum of 50 children to be vaccinated in any one center each a day, as an additional precaution in case of AEFIs. National vaccination rates of DPT dose and Hepatitis B dose sharply declined from 97% in 2012 to less than 60% in 2013 as a result of Quinvaxem suspension ^25^ and possibly parents’ hesitancy exposing to media information about AEFIs ^23^. Recently, some rural areas of Vietnam have also reported sporadic outbreaks of tetanus and diphtheria, indicating a potential gap in vaccine uptake ^26^. Additionally, our previous studies about vaccination barriers in these areas also revealed hesitancy about this EPI pentavalent vaccine from both local mothers and healthcare providers after exposing to related AEFIs news ^27^.

As internet individual usage have now reached 70 % for the Vietnamese population ^28^, people in Vietnam often look for online media for health information ^23, 29^. When AEFIs are reported, people may be more likely to search for vaccine information on the internet ^7^ thereby being exposed to various forms of information on different platforms. In Vietnam, amongst different types of media information, online newspapers are often considered more reliable and containing accredited sources of information resulting from a standardized process of production ^30, 31^. Thus, journalistic news reports, including digital forms, can have the potential to shape the public’s perspectives about important events ^31, 32^. However, the quality and accuracy of news reports, particularly about medical topics, may vary across sources as it is not subject to peer review, and journalistic processes do not always align with public health messaging ^33^.

Previous studies have indicated that exposure to media reports about adverse events related to vaccination can reduce Vietnamese parents’ vaccination intention for their children and lead to a decline in uptake ^23, 34, 35^. However, there is little information about the specific content of Vietnamese media, including news items, about vaccines. Understanding more about the form of information about vaccines accessed by the public is important, especially in the current context of the nationwide rollout of COVID-19 vaccinations, including more recently those for young children ^36^ and early media attention on adverse events ^37^. Given the large amount of press coverage of Quinvaxem and the significant decline in national vaccination rates around that time, we performed a study aiming at exploring characteristics of information and sentiments about this pentavalent vaccine in Vietnam online news.

## Methods

### Data collection

**Figure 1.**
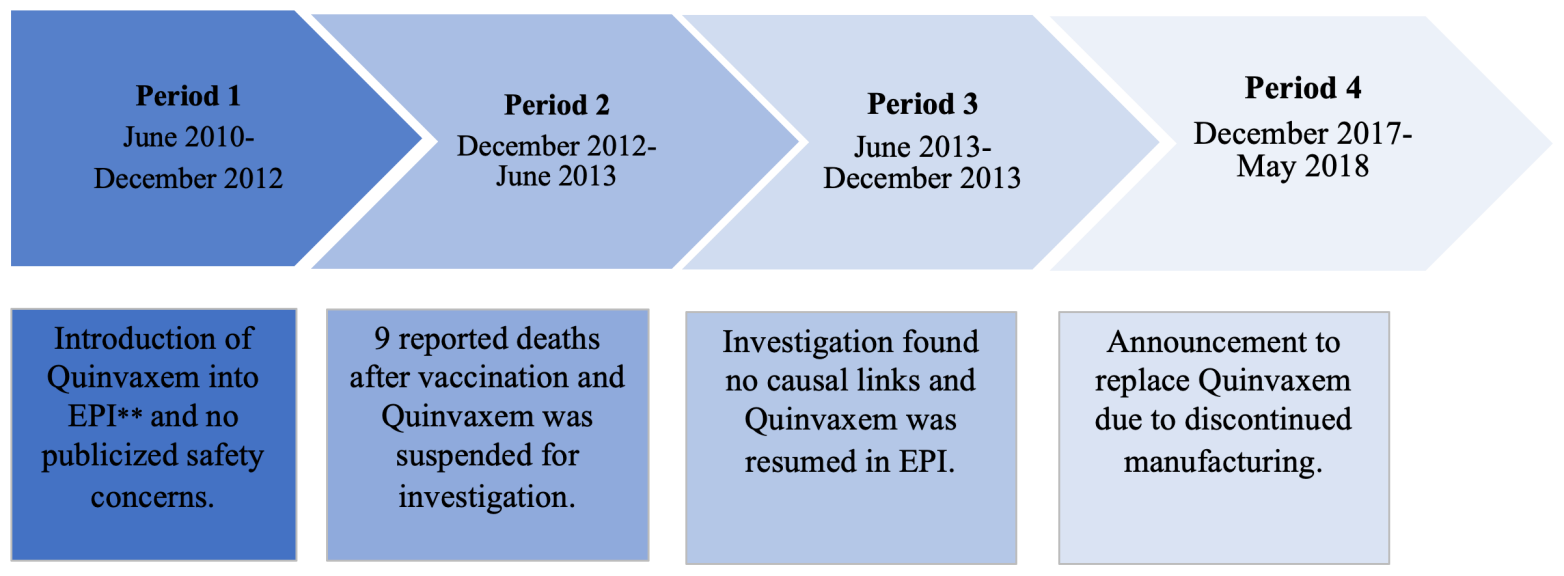
Four-time periods relating to the use of Quinvaxem vaccine in Vietnam. **EPI: the Expanded Program on Immunization.

We performed an Internet search using the Google search engine to identify news articles that reported information about the Quinvaxem vaccine. We searched for articles in four periods, between June 2010 (when Quinvaxem was introduced into the EPI) and May 2018 (when the MoH announced suspension of Quinvaxem from EPI). The four periods cover distinct periods relating to the reporting of AEFIs in the media (see Figure 1). The search was in Vietnamese using the following keywords Quinvaxem, 5-in-1 vaccine, Hib vaccine. Our search strategy included articles posted on official news platforms, including digital newspapers, magazines, or news compilation sites with at least 100 words about Quinvaxem ^38^. We excluded personal opinions found on social media platforms such as Facebook, Twitter, or internet forums. 

### Data analysis

We conducted content analysis in two steps: firstly, we prepared a quantitative summary of the article characteristics. This was followed by qualitative analysis for sentimental and emotional information. For the quantitative analysis, we developed a coding frame based on existing literature in order to categorize different article characteristics including main topic of the article, sentiments towards vaccine in the title and the content, and whether the article contains emotional tone ^39– 42^. We followed Habel and colleagues’ coding procedure in their study analyzing news content about the HPV vaccine ^40^. Two authors (HNT and CNTY) piloted the coding frame randomly on 10 articles and then discussed to refine the framework. To check for subjective assessment when coding, we assessed interrater reliability using kappa statistics with the acceptance threshold at 0.6 for moderate agreement ^40^. Both authors independently coded a 10% random sample (50 articles) and kappa values of interrater reliability ranged from 0.7 to 0.8, suggesting moderate to strong agreement. We had a further discussion to reconcile the remaining difference and finalized the coding frame ^43^ before the first author coded the remaining articles. All articles were numerated and entered into Microsoft Excel for quantitative analysis.

For qualitative analysis, we conducted in-depth content analysis to explore the themes of information that contain negative or positive sentiments and record specific emotional elements in all articles. We used the final coding frame of Step 1 to code all articles using Nvivo R1 (QSR International). We primarily coded for manifest content, meaning where the expression explicitly contains sentimental and emotional tone ^44^ (e.g. “ *the vaccine is unsafe*,” or “ *the incidence is very sad*”), but also considered context within where the vaccine information is written to code for latent sentiment ^44^ (e.g. *“Children die after vaccination: 55,000 shots were given”* coded for negative). Coded data were then read multiple times to identify the sentimental themes conveyed in the articles. Final results were reviewed by other authors.

### Ethical considerations

This study analyzed data collected from media articles that were in the public domain and therefore have not sought institutional ethical approval for the project. This paper is reported in line with the SRQR guidelines ^45^.

## Results

### Quantitative summary of article characteristics

In total, we found 452 articles and, after review for duplication and inclusion criteria, 360 articles were included in the analysis. These consisted of 7 articles published during Period 1, 98 articles from Period 2, 159 articles from Period 3, and 96 articles from Period 4. In general, the content of included articles was equally distributed into three topics: 142 (39%) concerned policy announcements, 127 (35%) concerned reporting of side effects reporting, and 91 (26%) concerned general information. Regarding sentiments towards this vaccine, most of the articles were neutral in their titles (255 articles, 71%) and content (249 articles, 60%) and did not contain emotion-conveying elements (271 articles, 75%). However, there were still 111 articles (31%) with mismatching sentiments between title and content, of which 37 articles (10%) had negative titles but neutral or positive content.

Table 1 details article characteristics for each period. Articles with contents reporting side effects accounted for approximately half of all articles published in Periods 1, 2, and 3, but none in Period 4. In Periods 2 and 3, the proportion of articles classified as containing negative content towards the vaccine were more than double those during Periods 1 and 4. The proportion of articles classified as conveying emotional content in Periods 1, 2, and 3 were between 30 and 43%, compared to only 5% in period 4. 

**Table 1.** Quantitative summary of Vietnam online news about Quinvaxem over four periods.

	Total		Period 1 *(Since Quinvaxem introduction* until first AEFI * reported)		Period 2 *(9 AEFIs* * reported)*		Period 3 *(Investigation* * period)*		Period 4 *(Announcement of* * replacing Quinvaxem)*	
	No	%	No	%	No	%	No	%	No	%
**Main topic of articles**										
General Information	**91**	**26%**	2	29%	20	20%	42	26%	27	28%
Policy announcements	**142**	**39%**	1	14%	25	26%	47	30%	69	72%
Side effects reporting	**127**	**35%**	4	57%	53	54%	70	44%	0	0%
**Sentiment in title**										
Positive	**20**	**6%**	1	14%	4	4%	15	9%	0	0%
Negative	**85**	**24%**	5	71%	32	33%	47	30%	5	5%
Neutral	**255**	**71%**	1	14%	62	63%	97	61%	91	95%
**Sentiment in content**										
Positive	**67**	**19%**	2	29%	10	10%	44	28%	11	11%
Negative	**78**	**22%**	1	14%	27	28%	38	24%	12	13%
Neutral	**215**	**60%**	4	57%	61	62%	77	48%	73	76%
**Sentiments in title and** ** content**										
Matched sentiment	**249**	**69%**	6	86%	71	72%	98	62%	74	77%
Different sentiment	**111**	**31%**	1	17%	27	28%	61	38%	22	23%
**Emotional tone**										
Yes	**89**	**25%**	4	57%	34	35%	47	30%	5	5%
No	**271**	**75%**	3	43%	64	65%	112	70%	91	95%
**Total**	**360**		**7**		**98**		**159**		**96**	

*AEFI: Adverse Events Following Immunization

**Figure 2.**
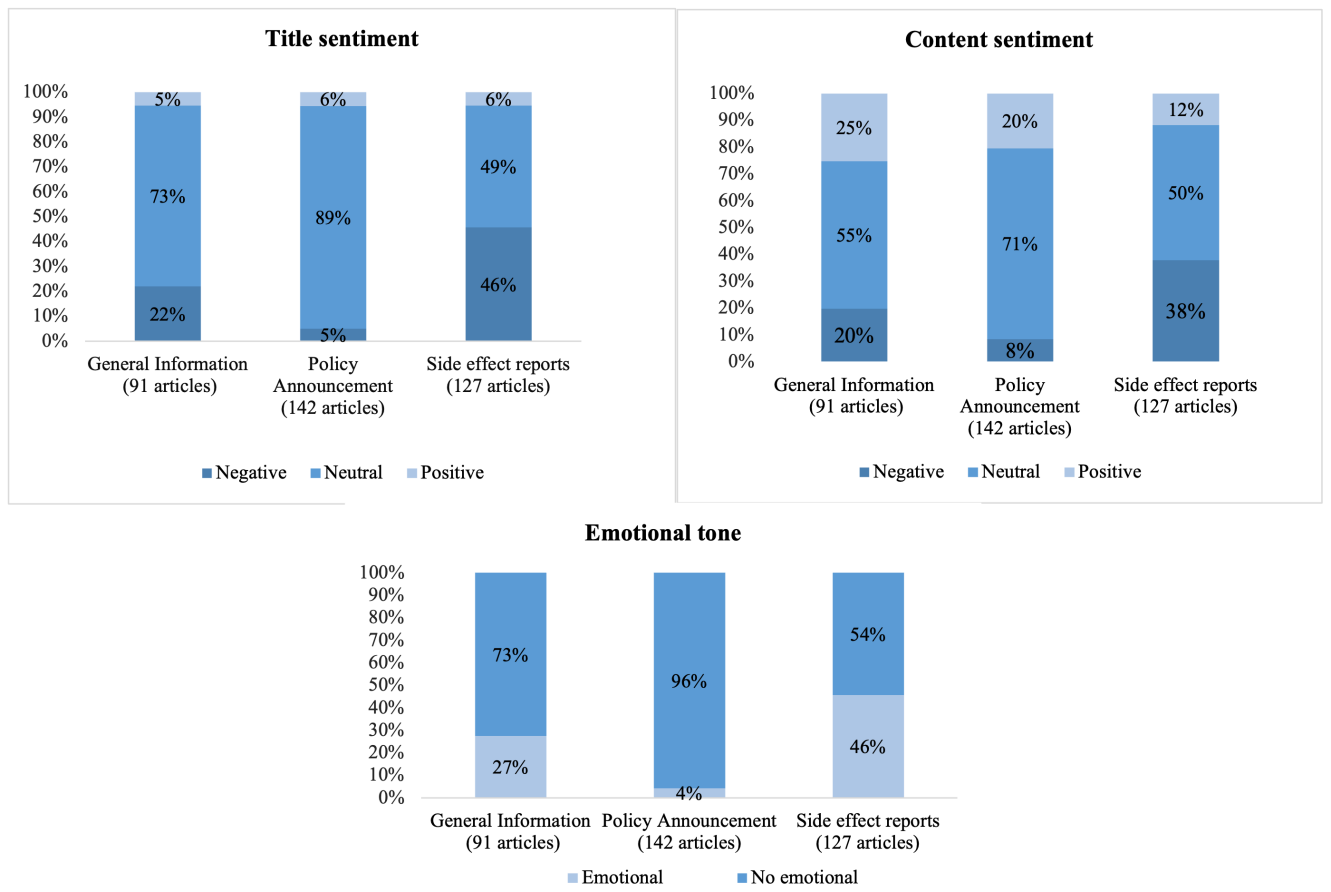
Sentiments and emotional tones in Quinvaxem articles with different content focus.

We also examined sentiments and emotional tone relating to article content ( Figure 2). Negative sentiment content was more often found in articles concerning side effects reporting than articles with general information or policy content. Overall, 49 (39%) of the articles reporting side effects had negative content, while only 18 (20%) of the articles concerning general information and 12 (22%) of articles concerning policy. In addition, 57 (45%) of the articles concerning side effects articles contained emotional elements compared to only 26 (28%) general information articles and 6 (4%) policy articles.

### Qualitative content analysis

Among 360 articles, we identified those with negative (155) and positive sentiments (148), and those which had emotive tones (68). Within these three categories, we identified themes as outlined in Table 2. We observed that sentiments were often expressed when discussing characteristics of the vaccine, healthcare practice or describing people’s attitudes and behaviors towards the vaccine. Table 2 lists the negative and positive themes with types of sentimental information within each theme.

**Table 2.** Content analysis of articles about Quinvaxem with negative or positive sentiments.

Themes	No. of articles (% of total 360 articles)
Negative themes	155 (43%)
Quinvaxem has problems with quality and safety • Low quality • Cheap • Old- generation vaccine so unsafe • High risk of severe reactions • Quinvaxem is the cause of mortality • There are AEFIs * related to Quinvaxem in other countries	102 (28%)
Health authority's decisions are “irresponsible” and questionable • Choosing vaccine based on low price over safety in EPI ** • Higher-income countries do not use Quinvaxem • Criticizing ways of communicating about side effects • AEFIs responses were slow • Decision to suspend Quinvaxem was abrupt and confusing • Using money to settle with families with AEFIs incidences • Investigation results are not persuasive • New policies about Quinvaxem continuation are controversial	60 (17%)
There are issues in the local implementation of vaccination • Problem with cold chain storage and facilities • Issues with health staff capacity • Local vaccination stations do not comply with standard procedure • Lack of consultation for parents • Health staff are worried and pressurized with Quinvaxem delivery	14 (4%)
People are anxious and hesitant about Quinvaxem vaccination • The community is anxious about Quinvaxem • Parents express refusal or delay of Quinvaxem vaccination for their children • Parents opt for an alternative, privately obtained vaccine • Healthcare independent experts object to continuing using Quinvaxem	61(17%)
Positive themes	148 (41%)
Quinvaxem is safe and of good quality • The vaccine had been assessed for meeting safety and quality standards before introduction in EPI • Quinvaxem is used in many other countries • Side effects are common and mild • Post hoc AEFI investigation reassures that the vaccine is safe • No evidence of a causal link between mortality and vaccines, including in other countries with similar AEFIs reports	93 (26%)
Quinvaxem contributes to public health efforts of preventing infectious diseases	60 (17%)
Local vaccination implementation meets standard requirements • Local vaccinators are qualified and experienced • Vaccination stations follow standard vaccination procedure • No evidence of a causal link between vaccination practice and AEFIs • Local practice was increasingly improved after AEFIs	43 (12%)
People show support towards Quinvaxem • Parents agrees vaccination is necessary to prevent diseases • Many parents bring children to vaccination • Parents encourage others to have children vaccinated • Parents feel relieved after AEFI investigation and improvement in procedure • Healthcare experts recommend vaccination	23 (6%)

*AEFI: Adverse Events Following Immunization **EPI: the Expanded Program on Immunization

***Negative sentiments towards the vaccine.*** Among 155/360 articles with negative sentiments about Quinvaxem, the most recurring negative theme was “Quinvaxem has vaccine quality and safety problems,” found in 102 articles ( Table 2). A significant focus of information in this theme was the high frequency of severe reactions of Quivaxem vaccine, including accusations of the vaccine being a direct cause of death, e.g., *However, in the past few months, this vaccine is considered the culprit taking away 9 children’s lives, in additions to dozens of other side effects”*- Article 73. Two other negative themes were related to healthcare practice, showing criticism towards health authorities’ decisions for important events (60 articles) and limitations in implementation practice at local vaccination stations (14 articles). The fourth theme, observed in 61 articles, cites attitudes of people about the vaccine which reflected different levels of vaccine hesitancy from parents and independent experts.

Looking across articles, we identified several issues with the content and wording of reports about side effects and AEFIs. There was an inconsistency in details of AEFIs among articles including differences in children’s names, incidence time, and the quantity and the types of side effects. For example, Article 74 says, *“Within half a month (20/12/2012-5/1/2013) there have been five fatalities (in children between 1-3 months old) following immunizations in Binh Dinh and Kien Giang provinces, and Hanoi”* while in fact, we were unable to find any reports of associated deaths in Binh Dinh province in this period. Additionally, many articles used misleading expressions about the vaccine and the Authorities’ responses to AEFI. For example, in articles in Period 3, typical side effects such as fever are also referred to as “adverse events” or “severe reactions” with exaggerated wording such as *“multiple”, “serial”, or “a mass of.”* In one case of AEFI, financial support for families with AEFIs was termed *“money to buy silence”* (so that the family will not take legal actions). Some articles did not include a balanced view of information, such as only including emotional narratives of the family about the event, omitting details about official response, or quoting ‘experts’ objection of the vaccine without giving their specific credentials.

***Positive sentiments towards the vaccine.*** For positive sentiments, we identified four themes in 148/360 articles ( Table 2). The most recurring theme claimed Quinvaxem to be safe and of good quality (93 articles), mentioning that the vaccine had been assessed for standard requirements by multiple organizations at different stages. These articles also provided reassurance regarding adverse events, e.g., side effects are mild, and reported mortality was coincidental. The second theme observed in 60 articles also relates to the characteristics of the vaccine, reporting the achievements of Quinvaxem and the EPI in reducing the prevalence of five diseases, e.g., *“Meanwhile, regarding benefits, Quinvaxem is very effective in reducing diphtheria by 410 times, and pertussis by 841 times.”-* Article 121. In the articles relating to healthcare practice, we only identified one theme regarding local vaccination implementation (43 articles). Information in this theme claims that local practice follows standard procedures and is not related to AEFIs. Articles with content of the fourth theme (23 articles) cited people’s supportive attitudes and behaviors about Quinvaxem, including both parents and healthcare professionals. 

***Emotional tone.*** We coded for three emotion-conveying elements in 89/360 articles. The other articles were considered neutral in tone. The most common feelings expressed were negative ones (70 articles), and included adjectives about sadness, worry and anger, and particularly words provoking sympathetic feelings for children as vaccine recipients. For example, *“It is so painful that Vietnamese children have to bear the risk of more reactions with this free-of-charge vaccine.”*- Article 247. In total, 31 articles contain punctuation marks in the title or content that conveys authors’ attitudes. For example, “ *Stop Quinvaxem immediately!* - “title of Article 60. Overall, 27 articles had emotional imagery, such as pictures of distressed children being vaccinated. These emotive patterns are frequently observed in information about side effects, comments about the Health Authority’s response, and specific narratives of AEFI incidents.

## Discussion

In general, this study indicates that adverse events following immunization is related to heightened attention of Vietnam’s online press towards the vaccine concerned. The amount of news coverage about Quinvaxem increased sharply from 7 articles in the first two-year period (before AEFIs) to 98 articles in the following six-months (when nine deaths were reported) and 159 in the six-month following AEFIs (investigation period). In the first three periods, approximately half of the articles focused their content on reporting side effects ( Table 1). In the qualitative findings, information about this topic was observed in almost all sentimental themes, most frequently in the vaccine safety and quality theme ( Table 2). Previous studies also observed discussion about this subject on other Vietnamese social media platforms in the same period ^23, 46^, illustrating that a variety of media are concerned with vaccine safety when AEFIs happen ^7^. Tsuda and colleagues ^18^ observed a similar increase in side effect reporting in Japanese news after HPV-related AEFIs, while other studies show that articles concerning side effects often attracted and influenced the audience’s perspectives more significantly than other content focus ^14, 21^. In this study, only in Period 4 – four years after first reported AEFIs when the MoH announced discontinuation of Quinvaxem, were there no articles primarily concerning side effects.

Most articles examined in this study presented neutral or positive sentiments towards Quinvaxem vaccine and had no emotional tone. However, content-wise, articles concentrating on side effects were coded for negative title/content sentiments and contained emotional expressions more often than articles with other focus ( Figure 2). Negative themes covered various factors blamed for AEFIs, ranging from vaccine safety to health policies to vaccine delivery to local vaccination stations, and provided quotes about people’s hesitant attitudes. It is documented that critical information about any of these aspects can decrease vaccine confidence and intention ^6, 9, 13, 23^. Adding emotional patterns to information can even amplify this ^20, 47^. Confirming other research on the use of emotional appeals in vaccine-related media, we also found similar patterns such as affective wordings, personal narrative of AEFIs, or distressing visualization ^12, 19^. What emerges from these patterns is that children are sometimes framed as vulnerable “victims” of vaccination and health policies. This is also seen in the anti-vaccination media ^19^. In addition, we found expressive punctuation in comments about vaccine safety and healthcare practice, such as exclamation marks or rhetorical question marks, used to intensify the feelings of frustration and suspicion. 

While side effects and AEFIs seem an appealing topic for the press, we identified conflicting information and misleading writing styles in many news stories, even with neutral- and positive- coded articles. For example, 37 articles (10%) had a negative title, but the contents shifted towards a balanced and, at time positive view about the vaccine. Although it may be common for journalists to use headlines to attract attention ^30^, this can initiate a false impression for the audience from the beginning and become even more unhelpful if readers just skim through the headings. The audience could become more confused and worried when looking up information of AEFIs in different articles as details of these incidents were inconsistently reported while common reactions were often termed as “adverse events” in exaggerated quantity expressions. Articles mentioning AEFIs from Quinvaxem and its temporary suspension in other countries notably to omitted details about the subsequent resuming of the vaccine after no causal evidence was found ^48^. Exaggeration, biased omission, unbalanced reporting, and sensationalization of events can negatively skew an audience’s perspectives ^20, 49^.

We also identified sentiment towards healthcare providers related to Quinvaxem vaccination as a major theme with both negative and positive expressions, suggesting the critical link in perception towards vaccine and healthcare policy and practice ^16^. Specifically, we observed a strong pattern of critical attitudes towards health authorities and their different policies related to Quinvaxem ( Table 2). The MoH’s decision to suspend this vaccine after AEFIs was critically reported to be abrupt and confusing, while other articles argued it was slow and irresponsible. Similar situations with HPV vaccine in Japan, Hepatitis B vaccine in China and Astra Zeneca COVID-19 vaccine in Europe showed that a suspension policy itself can decrease public vaccine confidence ^11, 21, 50^, negative framing of the decision in mainstream news can amplify anxiety. As the situation progressed, most subsequent articles added an explanation from the MoH in an attempt to balance the confusion. However, at the same time, other negative articles retrospectively questioned the decision to introduce the vaccine into the EPI in 2010. These distinct critiques raise the issue of trust in the ethics and competency of the health authority, which in turn can reduce readers’ acceptance towards government vaccination programs ^11, 22^. This is particularly important in the Vietnamese context, as communities in rural areas (>70% of the population) still largely rely on the free government EPI vaccines, whereas more wealthy segments of the population can reject them and access alternate vaccines at private clinics. In contrast, the COVID-19 vaccination is still solely delivered through the government, and trust in the authority can influence COVID-19 vaccine uptake.

While our qualitative analysis revealed that although there was positive information to balance views and promote the vaccine in juxtaposition with negative themes ( Table 2), it is arguable that this effort may have a limited impact on the audience. Even when readers tried to verify vaccine information through different sources, they would still find a confusing heterogeneity of information about vaccine safety and causes of AEFIs. Readers would also find it hard to validate what they read with official information. There were only 13 articles (4%) about the vaccines found in official newspapers of the MoH and Vietnam government. Where neutral/positive-coded media was mixed with reports containing harsh criticism towards health authorities, these ‘milder’ reports could be perceived as less certain. Moreover, opinions from healthcare experts in media towards Quinvaxem were reportedly divided with quotes of either encouragement or disapproval. The media also tended to highlight social hesitancy, with triple the number of articles citing people’s concerns about Quinvaxem compared to supportive pieces. All these media tendencies build a perplexing and unverifiable picture for an audience looking for information about Quinvaxem. According to Dixon and Clarke ^51^, such falsely- perceived balanced coverage of information can reinforce uncertainty and anxiety towards vaccines. In this situation, people may lower their vaccination intention over the uncontrollable immediate post-reaction risks, particularly when disease prevalence is low ^51– 54^. We also observed negative and challenging comments from readers responding to some neutral- and positive- coded articles. For example, under Article 33, coded positive for both title and content for reassuring no causal link between Quinvaxem and AEFIs, all readers’ comments expressed strong suspicion towards this claim and health authorities, e.g *.,” How come they [National Institute of Hygiene and Epidemiology] said it’s not related when children died of vaccination? Would these children still have died if they had not been vaccinated”* posted by a public reader.

### Limitations

This study has several limitations. As a retrospective study, we may have missed articles whose hyperlinks had expired or been deleted. However, as included articles were close to the timeline of important events related to Quinvaxem ( Figure 1) and retrieved from a wide range of sources, we are confident that these articles still reflected news coverage about the vaccine at that time. Besides, for the purpose of exploring news around important events related to Quinvaxem, we did not include articles from 2014-2017 when the vaccine was routinely given without sensational news coverage. This prevented us from drawing a comprehensive longitudinal analysis of the trend of information. We also did not screen social media or internet forums and could not assess the link between news coverage and social media activity. This study mainly focuses on the description of the news content, so we were unable to draw a firm conclusion about readers’ reception of the articles except where they posted comments to online articles. 

### Implications for future vaccine communication

This study has important implications for vaccine and public health communication. First, it highlights the ethical issue of balancing the pressure to attract readers and high-quality reporting in medical journalism. The competitive nature of the media industry can drive journalists to use more click-bait titles and sensational writing styles ^55, 56^, as reflected by the shortfalls in published articles about Quinvaxem, such as detail inaccuracy, emotional language and punctuation, and unbalanced coverage. However, media creators may have inadvertently underestimated the severe and far-reaching consequences of their negative portrayal of vaccine topics on individual and population health. Media coverage about AEFIs could contribute to delaying or refusing vaccination at that time ^15^, which can entail a latent risk of future infections without booster efforts ^8^. Moreover, past information about post-reactions may still be exploited by anti-vaccination communities years later, as seen in the case of the MMR vaccine-autism debate ^53, 57^. Recent studies also pointed out how media reports of rare AEFIs related to COVID-19 vaccines have fueled vaccine hesitancy in different places ^5, 9^, dampening global vaccination delivery and pandemic response efforts. Hence, it raised the need to improve social responsibility of media creators not only at the individual journalist level but also at editorial management when writing medical stories, including vaccination. More neutral and accurate portrayals of public health events also aligns with Vietnam’s national strategy to require the press to be objective and constructive in reporting impactful social issues ^58^.

Our findings also suggested that the authorities, medical experts, and media creators need to collaborate in producing and monitoring vaccine media. With unregulated social media, it will be impossible to remove all fake and negative news, but a strong collaboration among these stakeholders could maximize the positive influence of accurate coverage, as has been the case of COVID-19 communication in Vietnam. During the pandemic, the government and the Ministry of Health established different communication channels and strictly regulated public information about the pandemic ^59^. There were strict punishments for circulating fake news on any media platform, and misleading and negative media was quickly removed (Decree 15/2020/NĐ-CP, 2020). The strong communication strategy in Vietnam is considered as one of the most effective components of the largely successful national response to the pandemic in 2020 ^59^. Continuing such collaborative communication is also of pertinent importance to Vietnam at the moment when the government has started COVID-19 vaccination for younger children, and digital news has reported some cases of related AEFIs in teenagers ^37^. Future studies are needed to explore Vietnam’s media coverage of COVID-19 vaccines and its link with vaccine confidence in comparison with the Quinvaxem vaccine to picture a clearer understanding of the ongoing public impact of vaccine journalism in Vietnam. Above all, we strongly recommend that a corroborative and timely effort similar to that deployed for COVID-19 communications should be applied to the delivery of all essential vaccines in Vietnam.

## Conclusion

In summary, our study contributes to the overall literature about vaccines in media, showing that adverse events following immunization of a childhood combination vaccine can lead to an upsurge in news coverage. However, we observed conflicting and even exaggerated details across reports about AEFIs incidents and vaccine side effects in Vietnam online news. Content analysis of screened articles revealed that negative sentiments were often found in articles reporting side effects. These articles questioned vaccine safety and criticized health authority, accompanied by expression of mainly negative feelings such as sadness, fear, and doubt. Despite the presence of positive and neutral articles in juxtaposition, the heterogeneity of information across news channels may reinforce uncertainty about vaccine safety and decrease readers’ vaccine intention. Given the current rollout of COVID-19 vaccination for younger children in Vietnam, these findings can be helpful to inform communication strategy for vaccine messaging, which should incorporate improving social responsibility for journalists and enhancing editorial supervision on vaccine content. Through this study, we call for a stronger collaboration between media creators, medical experts, and the government in Vietnam in communicating about vaccines to maintain public trust in vaccination.

## Data Availability

Dryad: Media Paper Data: https://doi.org/10.5061/dryad.mkkwh713k ^43^ This project contains the following underlying data: List-of-articles-Media-paper.xlsx (List of article names, publication date, and webpages where articles were retrieved to be included in the study) Dryad: Media Paper Data: https://doi.org/10.5061/dryad.mkkwh713k ^43^ This project contains the following extended data: Coding-framework-for-vaccine-media-articles.xlsx (Coding framework to classify articles into the following categories: the main content of the article, title sentiment, content sentiment, and emotional tone) Data are available under the terms of the Creative Commons Zero "No rights reserved" data waiver (CC0 1.0 Public domain dedication). Zenodo: SRQR checklist for manuscript “Media portrayal of vaccine - a content analysis of Vietnam online news about a pentavelent vaccine in the EPI”. https://doi.org/10.5281/zenodo.7179651 ^45^. Data are available under the terms of the Creative Commons Attribution 4.0 International license (CC-BY 4.0).
