# Supplementary material for: Media portrayal of vaccine: a content analysis of Vietnam online news about a pentavalent vaccine in the Expanded Program of Immunization
Source: Wellcome Open Res. 2022 Nov 2;7:271. [Version 1] doi: 10.12688/wellcomeopenres.18457.1 (PMC11362720; doi:10.12688/wellcomeopenres.18457.1)
Supplement: Peer review history

# Reviewer report: Reviewer response for version 1

**DOI:** 10.21956/wellcomeopenres.20468.r53203

**Published:** 2024-08-29

**Authors:** Atsuyoshi Ishizumi

**Affiliations:** 1 US Centers for Disease Control and Prevention, Atlanta, GA, USA; 2 The Task Force for Global Health, Decatur, Georgia, USA

This study characterizes how the media portrayed the roll-out of Quinvaxem and its associated AEFIs across four different time periods in Vietnam. Data collection and analysis appear to have been performed in accordance with established methods.

 Although this study is based on data and events that are several years old, its findings and implications are still relevant especially considering the importance of the vaccine information ecosystem in the context of COVID-19. I just have a few minor clarifying questions/comments.

- For the quantitative summary of included articles, what exactly does the topic "general information" consist of? Perhaps helpful to provide a few examples?
- For search strategy and inclusion criteria: did you include press releases from public health authorities? You state that "official newspapers of the MoH and Vietnam government" were included but wasn't sure if this included press releases. 
- I think you allude to this in the discussion section when you discuss social media, but to further strengthen this section, you could also briefly frame your findings in the context of the broader information ecosystem. For many consumers, news media is only one of the many information sources that comprise their overall information diet.
  Furthermore, there are other factors that can potentially shape how people consume or interpret news content, such as information literacy, presence of fact-checkers, their sharing behaviors, etc. I understand that these topics are not within the scope of your study, but it might be helpful to touch on these other factors especially where you discuss how exposure to news content could potentially reinforce uncertainty about vaccines or fuel vaccine hesitancy.

Is the work clearly and accurately presented and does it cite the current literature?

Yes

If applicable, is the statistical analysis and its interpretation appropriate?

Not applicable

Are all the source data underlying the results available to ensure full reproducibility?

Yes

Is the study design appropriate and is the work technically sound?

Yes

Are the conclusions drawn adequately supported by the results?

Yes

Are sufficient details of methods and analysis provided to allow replication by others?

Yes

Reviewer Expertise:

Vaccine confidence and demand, behavioral and social drivers of vaccination, infodemic management for immunization programs, qualitative thematic analysis

I confirm that I have read this submission and believe that I have an appropriate level of expertise to confirm that it is of an acceptable scientific standard.

# Reviewer report: Reviewer response for version 1

**DOI:** 10.21956/wellcomeopenres.20468.r68692

**Published:** 2023-11-21

**Authors:** Basema Saddik

**Affiliations:** 1 University of Sharjah, Sharjah, Sharjah, United Arab Emirates

This is a very interesting paper that provides a comprehensive quantitative and qualitative analysis of news articles related to the Quinvaxem vaccine in Vietnam. The combination of both approaches enhances the richness and depth of the findings which shed light on the complex interplay between media portrayal, public perception, and vaccine confidence. 

 I have added some additional comments which could help improve the manuscript.  

**Introduction:**

1. In the first paragraph, the authors refer to the Arce *et al. *paper stating that LMICs report higher acceptance rates compared to the USA and Russia, motivated by interest in personal protection. It is not clear here whether the higher or lower acceptance rates are motivating personal protection. Perhaps rephrase to make it clearer.
2. 4th paragraph, Line 7: remove "a" between each and day.
3. 4th paragraph, last line: change "after exposing" to "being exposed".
4. 5th paragraph, first line: replace "have" with "has".
5. Throughout the introduction, the authors refer to the nationwide rollout of COVID-19 vaccinations. Is this part of a new scheduled vaccination program or an extension of a previous rollout that occurred during COVID-19? It would be beneficial to provide more context about this.

**Methods:**

1. Overall the methodology described by the authors is comprehensive and the combination of the quantitative and qualitative methods, along with the reliability testing undertaken strengthens the validity and reliability of the content analysis. It would be useful if the authors could briefly describe the Habel and colleagues coding procedure used to give the reader context of the information extracted. Additionally, it is mentioned in the data collection that the search was in Vietnamese and I am assuming the method/coding procedure used (Habel and colleagues) was in another language. How was the translation done?
2. It is not clear whether a triangulation method was used to validate qualitative findings. Please clarify this. 
3. In the final paragraph of the methods section, the authors state that coded data were read multiple times to identify the sentimental themes. Could there be a potential bias in using this method such as the subjective nature of sentiment analysis? Consider addressing how the researchers mitigated these biases and the limitations associated with this method. 

**Discussion:**

1. Paragraph 3: This statement, "Articles mentioning AEFIs from Quinvaxem and its temporary suspension in other countries notably to omitted details about the subsequent resuming of the vaccine after no causal evidence was found" is not very clear. Perhaps rephrase. 
2. The authors mention the strict government punishments used for circulating fake news during the pandemic. Are the rules more lenient in other vaccine media? Perhaps elaborate on this further in the discussion and how this could contribute to more reliable coverage. 
3. Was there any consideration for temporal factors? As the authors have mentioned, news and sentiments can change over time, so acknowledging the temporal context may add depth to the interpretation of results. The study acknowledges its focus on specific time frames related to sensational news coverage. However, a more detailed temporal analysis, especially during the routine administration of the vaccine (2014-2017), could provide a more comprehensive understanding of media trends over time.
4. Although the authors have outlined the limitations, they could further elaborate on the potential biases introduced by the exclusion of certain articles, potential sources of bias in the data, and steps taken to mitigate these biases.

Is the work clearly and accurately presented and does it cite the current literature?

Yes

If applicable, is the statistical analysis and its interpretation appropriate?

Partly

Are all the source data underlying the results available to ensure full reproducibility?

Yes

Is the study design appropriate and is the work technically sound?

Yes

Are the conclusions drawn adequately supported by the results?

Yes

Are sufficient details of methods and analysis provided to allow replication by others?

Partly

Reviewer Expertise:

Professor of Epidemiology and Public Health

I confirm that I have read this submission and believe that I have an appropriate level of expertise to confirm that it is of an acceptable scientific standard.
